# Ultra High-Resolution *In vivo* Computed Tomography Imaging of Mouse Cerebrovasculature Using a Long Circulating Blood Pool Contrast Agent

**DOI:** 10.1038/srep10178

**Published:** 2015-05-18

**Authors:** Zbigniew Starosolski, Carlos A. Villamizar, David Rendon, Michael J. Paldino, Dianna M. Milewicz, Ketan B. Ghaghada, Ananth V. Annapragada

**Affiliations:** 1 Edward B. Singleton Department of Pediatric Radiology, Texas Children’s Hospital, Houston TX; 2 Department of Radiology, Baylor College of Medicine, Houston, TX; 3 Department of Internal Medicine, University of Texas Health Science Center at Houston, Houston, TX; 4 Department of Pediatrics, Baylor College of Medicine, Houston, TX

## Abstract

Abnormalities in the cerebrovascular system play a central role in many neurologic diseases. The on-going expansion of rodent models of human cerebrovascular diseases and the need to use these models to understand disease progression and treatment has amplified the need for reproducible non-invasive imaging methods for high-resolution visualization of the complete cerebral vasculature. In this study, we present methods for *in vivo* high-resolution (19 μm isotropic) computed tomography imaging of complete mouse brain vasculature. This technique enabled 3D visualization of large cerebrovascular networks, including the Circle of Willis. Blood vessels as small as 40 μm were clearly delineated. *ACTA2* mutations in humans cause cerebrovascular defects, including abnormally straightened arteries and a moyamoya-like arteriopathy characterized by bilateral narrowing of the internal carotid artery and stenosis of many large arteries. *In vivo* imaging studies performed in a mouse model of *Acta2* mutations demonstrated the utility of this method for studying vascular morphometric changes that are practically impossible to identify using current histological methods. Specifically, the technique demonstrated changes in the width of the Circle of Willis, straightening of cerebral arteries and arterial stenoses. We believe the use of imaging methods described here will contribute substantially to the study of rodent cerebrovasculature.

The cerebral vascular network plays a critical role in many brain pathologies, including ischemic and hemorrhagic strokes, neurodegenerative diseases, and brain tumors^1^,^2^. With the ability to engineer mouse models of human diseases, non-invasive 3D imaging of cerebrovasculature in rodent models is a critical tool needed to study the role of the blood circulatory system in brain pathologies^3^. While a variety of imaging modalities have been used for interrogation of rodent cerebrovasculature, they are all limited either in their spatial domain or in their spatial resolution. There is an imminent need to develop a simple and robust imaging technique that provides reproducible and quantitative assessment of vascular morphology at high spatial resolution, through the entire cerebrovascular system. Such a tool would also facilitate a standardized and universal approach for studying and comparing new data generated in rodent models. Finally, the ability to perform such studies in live animals would enable physiologically realistic studies, as well as potentiate longitudinal studies, which are vital for the evaluation of therapeutic interventions.

Magnetic resonance imaging (MRI) has been used to study the role of vasculature in Alzheimer’s disease and brain tumors in rodent models^4^,^5^,^6^,^7^. Ultra-high resolution MRI (≤ 50 μm isotropic) has been investigated using a combination of high field MR magnets, specialized coils and extended periods of image acquisition^7^,^8^,^9^. Optical techniques such as intravital microscopy, using two- and three-photon microscopy, have been utilized to assess sub-surface changes at ultra-high spatial resolution, but only over a relatively small area^10^,^11^,^12^. This technique is limited to blood vessels at the surface of the brain because of limited penetration depth of imaging and, therefore, does not enable visualization of vasculature throughout the brain. It also usually requires a cranial window to be installed, complicating the study. Histological imaging of arteries serves as the gold standard for ultra-high resolution assessment of vascular pathologies, however the technique is limited to *ex vivo* analysis and is not suitable for macroscopic analysis of vascular morphometry over the entire brain circulation, or for longitudinal studies. Nuclear imaging modalities (micro-PET and micro-SPECT) have not been used for high-resolution vascular imaging due to the intrinsic physics limitations on reconstructed spatial resolution of PET and SPECT scanners. However these techniques provide valuable functional information in the setting of vascular pathologies^13^,^14^.

X-ray based techniques such as computed tomography (CT) have been evaluated for imaging of live animals^15^,^16^. Unlike MRI, X-ray imaging techniques are flow independent and therefore issues of non-uniform signal intensity and vessel visibility, caused by variable blood flow, are automatically overcome. These are critical requirements for quantitative vascular morphometry because poor vessel visibility leads to key vessels not being assessed in a morphometric analysis, and thus skewing the results. Contrast-enhanced CT imaging enables 3D interrogation of vasculature at high spatial resolution^17^,^18^,^19^. High-resolution CT imaging in small animals, however, requires long scan times (>20 min) to obtain images with sufficient signal to noise ratio (SNR)^20^,^21^. As a result, conventional iodinated contrast agents, which have very short blood half-life, are not suitable for such long-acquisition times. We demonstrate here that this limitation of conventional contrast agents can be overcome using long circulating blood pool contrast agents. Several macromolecule and nanoparticle-based platforms have been investigated for the development of long circulating CT contrast agents^22^,^23^. Contrast agents based on iodine^24^,^25^,^26^,^27^, gold^28^,^29^ and bismuth^30^,^31^ have been extensively investigated for use in in vivo CT imaging. These agents are cleared slowly from the circulation and thus enable uniform and stable opacification of the entire vascular system.

In this study, we present the development and optimization of a technique for ultra high-resolution imaging of the complete mouse cerebral vasculature using micro-CT and a blood-pool liposomal-iodinated contrast agent. Beam energy, photon flux, related scan parameters and contrast agent dose are all shown to impact vessel visibility and overall image quality, but also the associated radiation dose, potentially impacting the validity of longitudinal studies. The techniques presented here enable imaging of the complete mouse cerebral vasculature with exquisite detail, while minimizing radiation dose.

The technique was used to study the cerebral arterial system in a mouse model of cerebrovascular anomalies. Mutations in *ACTA2* cause cerebrovascular disease characterized by straightening of cerebral arteries and a moyamoya-like pattern of distal occlusions of the internal carotid arteries and stenosis of arteries within the Circle of Willis^32^,^33^. The imaging technique was used to assess mice deficient in *Acta2* (*Acta2*^*+/-*^ and *Acta2*^*-/-*^)^34^ and we found straightening of cerebral arteries and morphometric changes in the width of the Circle of Willis in the *Acta2* deficient mice when compared with the wild type mice. Additionally, arterial stenotic events were observed, lesions that are difficult to capture by conventional techniques. Thus, the imaging technique presented in here is a simple, reproducible and quantitative approach for performing complete cerebral vascular morphometry at high spatial resolution in live animals.

## Materials and Methods

Animal studies were performed under a protocol approved by the Institutional Animal Welfare Committee (IACUC) of the Baylor College of Medicine. The studies reported in this paper are in accordance with the NC3RS ARRIVE guidelines.

A total of 51 male C57BL6/J mice (20–28 g) were used for the method development study. The utilization of the technique, to delineate cerebrovascular pathologies, was performed in a total of 22 mice (seven wild type (WT), nine heterozygous (*Acta2*^*+/-*^) and six knock-out (*Acta2*^*-/-*^)) imaged at sixteen weeks of age. A custom-designed 3D printed mouse head holder, modified from one used previously for mouse imaging^5^, was used to improve scan volume reproducibility and reduce animal motion during image acquisition. In preliminary studies, we found that anesthesia was sporadically unstable, and even at relatively high levels of isoflurane (~2.5-3%), animals would show signs of awakening, characterized by motion in the scanner, and consequent blurred images. This instability was traced to poor anesthetic gas delivery, and we hypothesized that the positioning of the mouse on the scanner bed resulted in partial airway obstruction, and positioning the mouse differently, in a manner reminiscent of the technique used in cardio pulmonary resuscitation: head tilt-chin lift (Supplementary Figure 1 and 2) would improve the airflow. The following refinements were therefore made to the design of the bed: (1) outer shape refinement to match the scanner’s standard mouse bed; (2) reorientation and modification of contact surface for nasal bone and premaxilla, (3) additional outlet for ventilation; (4) re-designing of bite bar and addition of bite bar clip to secure the animal in a position that assured an open airway. The model was in-house 3D printed with a fusion deposition modeling (FDM) printer with a spatial accuracy of 0.2 mm. Acrylonitrile butadiene styrene (ABS) was selected as the material for 3D printing due to its durability and low X-ray attenuation. The use of this holder greatly reduced animal motion during image acquisition.

A long circulating liposomal-iodinated contrast agent that is cleared slowly from the blood was used for contrast-enhanced studies. The liposomal contrast agent was prepared using methods described previously^35^. The liposomes in the final product solution had an average particle size of 140 nm with a polydispersity index less than 0.15. The final iodine concentration of the liposomal contrast agent was 110 mg I/mL.

### Micro-CT setup

CT angiography was performed on a commonly available, small animal micro-CT system (Inveon, Siemens Inc., Knoxville, TN, USA). The manufacturer reported maximum effective resolution is ~15 μm depending on magnification factor, binning settings and individual scanner calibration (Reference: Siemens User Manual). Performance evaluations for this micro-CT scanner have been reported previously^36^,^37^. The animals were scanned while free breathing under anesthesia using 1.5 – 2.5% isoflurane delivered by face-cone. An electrical resistive heating element was used to maintain and control body temperature during the entire imaging session. The respiratory rate was monitored using a pressure-pad placed under the animal in the abdominal region. The contrast agent was administered over a 1-2 minute period using a manual syringe and a catheter placed in the tail vein.

Image quality and vessel visibility were studied as a function of the following parameters: peak voltage setting (50 kVp and 70 kVp), voxel size (19 μm and 35 μm), number of image projections (360, 720, 1440) and contrast agent dose (1.1 and 2.2 g Iodine/kg) (Supplementary Table 1). These four parameters were selected to cover the range of variables available to the general micro-CT user, and have a direct effect on image quality and radiation dose. The number of projections, which were used in image reconstruction, enabled a study of the impact of radiation dose on image quality; minimizing the dose is desirable in longitudinal imaging studies. The magnification factor, which defines voxel size and spatial resolution, was varied by changing the distance between the source and the subject. In this study, images acquired at 19 μm isotropic voxel size will henceforth be referred to as high-resolution (*high-res*) images; images acquired at 35 μm isotropic voxel size will be referred to as low-resolution (*low-res*) images. The source-to-detector (SD), source-to-center-of rotation (SCR) and geometric magnification factor were 309.90 mm, 98.43 mm and 3.15, respectively, for high-res scans and 312.93 mm, 183.93 mm and 1.70, respectively for the low-res scans.

The following parameters were kept constant for all the scans: 850 ms x-ray exposure, 0.5 mA tube current and 1440 image projections. A long x-ray exposure window was used to generate sufficient photon flux, to compensate for the relatively low tube current specifications on the micro-CT scanner (0.5 mA is the maximum setting). A large number of image projections were acquired to obtain adequate SNR compensating for the relatively small voxels (~6.9 × 10^−6 ^μL at 19 μm isotropic voxel size). This resulted in an average scan time of 52 minutes. The acquired X-ray projection images were reconstructed into 3D datasets using a filtered back-projection reconstruction algorithm on Cobra software (version 6.3.39.0, EXXIM Computing Corporation, Pleasanton, CA, USA). The image datasets were reconstructed using 1440, 720 or 360 projections to assess the impact of projection number on image quality. All datasets were Hounsfield Units (HU) calibrated for image analysis.

The incident radiation dose associated with various peak voltage and voxel size was determined using a radiation monitoring device (Radiation Monitor model 2026C coupled with a model 20 × 6-0.6 Electrometer/Ion sensor; Radcal Corporation, Monrovia, CA, USA). Radiation dose estimation scans were performed while placing the sensor at the center of the field of view. Scans were performed with 180 acquired projections at each combination of peak voltage and voxel size. The dose obtained for these scans was then scaled to the 360, 720 and 1440 projection scan protocols using scaling factors of 2, 4 and 8, respectively. The radiation dose ranged from 0.54 Gy (50 kVp, 360 projections) to 13.07 Gy (70 kVp, 1440 projections) (Supplementary Table 2).

### Data Analysis

Analysis of images was performed in Osirix (version 5.8.5 64-bit; Pixmeo, Bernex, Switzerland), ImageJ (version 1.48; NIH, Bethesda, MD, USA) and MATLAB (version 7.13; Natick, MA, USA). Signal attenuation, measured in Hounsfield Units (HU), was determined in one artery (internal carotid artery, ICA) and one vein (great cerebral vein of Galen, GVG) located within the brain (Supplementary Figure 3). SNR calculations were performed in both these vessels. Circular regions of interest (ROIs) were drawn in the target blood vessel and nearby brain parenchyma (control background region). For SNR analysis, standard deviations of ROIs drawn within the brain parenchyma were used as noise levels.

The diameters of major vessels within the Circle of Willis were determined using a semi-automatic approach. First, vessel enhancement was performed in ImageJ using the Ferangi filter. Then, vessel segmentation and skeletonization were performed to obtain the centerlines of each vessel. Target vessel structures were manually selected and the radial cross-section lengths of all vessel segments were determined using a Matlab routine. Vessel diameter was calculated as a median of the set of minimal radial lengths of all connected vessel segments.

Quantitative analysis was performed on three vessel networks that collectively covered the majority of the brain volume: (1) pontine arteries, (2) transverse hippocampal arteries, and (3) left middle cerebral artery (MCA) branches. Their small size (pontine), proximity to the skull (MCA) and radial orientation (hippocampal arteries) provided a metric for evaluating the robustness of vessel delineation using our imaging technique. The number of pontine arteries, originating from a 3.0 mm length of basilar artery (measured from its origin at the convergence of the ventral arteries) was determined. A similar process was implemented for the analysis of transverse hippocampal arteries and left MCA branches. A 4-point scale was used to characterize visibility of vascular structures: Absent-0; weak-1; good-2; very good-3.

Vascular morphometric analysis was performed on multiple cerebrovascular structures in wild type, *Acta2*^*+/-*^ and *Acta2*^*-/-*^ mice. The Circle of Willis was divided into multiple vessel segments with the origin defined at the intersection of the internal carotids arteries and the Azygos Pericallosal artery. The width of the Circle of Willis was then determined as the maximum distance between corresponding vessel segments (Supplementary Figure 8). Vessel straightening was evaluated by determining the middle curvature and the arc:length ratio. A detailed description of all the vascular morphometric parameters is provided in the supplementary section.

### Statistical Analysis

Analysis of statistical significance was done without assumption of normal distribution. Kolmogorov–Smirnov non-parametric statistical testing was performed to evaluate normality hypothesis of data distribution. P-values were calculated with a two-sided t-test if the data were normally distributed. In all other scenarios, statistical analysis was conducted using the two-sided Wilcoxon Rank-Sum test.

The effect of protocol parameters i.e., peak voltage (kVp), voxel size (spatial resolution) and contrast agent dose on calculated values of mean CT signal within regions of interest, vessel diameters and visibility score were examined. The detailed results of statistical analysis are provided in the supplementary information.

## Results

**Figure 1:**
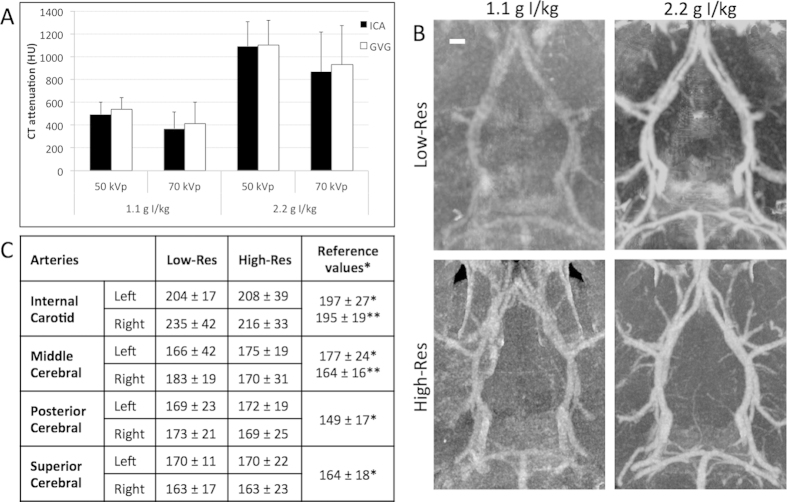
Effect of contrast agent dose and spatial resolution on visualization of the Circle of Willis. (**A**) Vascular CT signal as a function of contrast agent dose and peak voltage. Analysis is performed in the internal carotid artery (ICA) and great cerebral vein of Galen (GVG). The differences in vascular CT attenuation obtained using a contrast agent dose of 1.1 g I/kg and 2.2 g I/kg are statistically significant for ICA and GVG at either peak voltage setting (p < 0.01). Mean background brain tissue CT signal was 51 HU for 1.1 g I/kg and 50 kVp, -87 HU for 1.1 g I/kg and 70 kVp, 73 HU for 2.2 g I/kg and 50 kVp and -20 HU for 2.2 g I/kg and 70 kVp. (**B**) Coronal thick slab maximum intensity projection images demonstrating the mouse Circle of Willis as a function of contrast agent dose and voxel size. The skull is segmented and subtracted out for better visualization of the mouse Circle of Willis. Scale bar represents 500 μm. (**C**) CT imaging-derived diameters of major Circle of Willis arteries determined using low-res and high-res scan protocols. Vessel diameter (μm) is expressed as mean ±SD. The differences in the vessel diameter obtained using either of the protocol is not statistically significant (p < 0.05). Analysis was performed on images acquired using a contrast agent dose of 2.2 g I/kg. Literature reported vessel diameters are provided in the final column (^*^ - ref^38^; ^**^ -ref^39^).

The administration of liposomal-iodinated contrast agent results in high vascular CT attenuation (Fig. 1A). Vascular signal intensity increase with contrast agent dose and the increase with contrast dose is identical in both arterial and venous structures. The peak voltage setting does not significantly impact vascular CT attenuation at either of dose levels. Additionally, contrast agent dose and image spatial resolution have independent effects on image quality. Image quality is substantially worse at the low contrast agent dose (1.1 g I/kg), and low-res scans with low contrast agent dose yield the poorest images (Fig. 1B). At a high dose of contrast agent (2.2 g I/kg), all the major arteries within the Circle of Willis are clearly visualized. Vessel diameters of major arteries within the Circle of Willis were determined using low-resolution and high-resolution images (Fig. 1C). Image resolution (voxel size) does not have a significant impact on the measured diameters of these arteries, suggesting that the lower resolution measurements are sufficient for accurate diameter analysis of large arteries as long as the images are acquired with high dose of the contrast agent (Fig. 1C).

**Figure 2:**
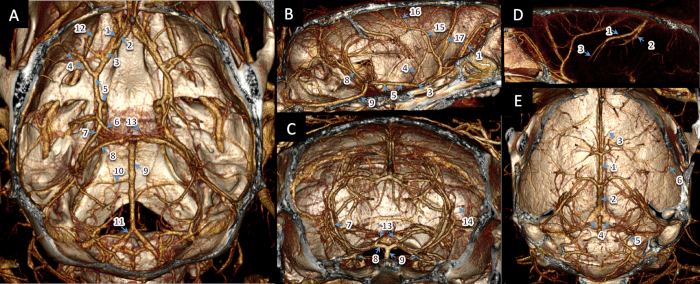
Volume-rendered 3D images of the cerebral vasculature in mice. Panels (A-C): (1) Olfactory artery (OlfA), (2) Anterior communicating artery (AcomA), (3) Anterior cerebral artery (ACA), (4) Middle cerebral artery (MCA), (5) Internal carotid artery (ICA), (6) Posterior communicating artery (PcomA), (7) Posterior cerebral artery (PCA), (8) Superior cerebellar artery (SCA), (9) Basilar artery (BA), (10) Anterior inferior cerebellar artery (AICA), (11) Vertebral artery, (12) Ophthalmic artery, (13) Artery of Percheron, (14) Transverse hippocampal arteries; (15) Azygos pericallosal artery (AzPa), (16) Posterior internal frontal artery; (17) Medial orbitofrontal artery. Panels (D-E): (1) Great cerebral vein of Galen, (2) Longitudinal hippocampal vein, (3) Thalamostriate vein, (4) Transverse sinus, (5) Sigmoid sinus, (6) Long cortical branch. Images (high-res) were acquired using the following parameters: 50 kVp, 1440 projections, 19 μm isotropic voxel; 2.2 gm I/kg contrast agent dose.

In addition, sub-100 μm vessels, such as the posterior communicating artery (PcomA), thalamic arteries, transverse hippocampal arteries (THA) and pontine arteries (PA), are clearly visualized in the high-res images (Fig. 2). Small and large venous vessels are also clearly demonstrated. Pontine vessels, as small as 40 μm, are clearly visible only in the high-res images acquired at 19 μm voxel size ([Fig f3]) (Please also refer to supplementary movie). Key requirements for obtaining such high-resolution (19 μm isotropic voxel) images include long scan times (~ 50 minutes) enabling the collection of a large number of projections (upto 1440 projections acquired over a 360° angle) and stable opacification of the blood pool during this long image acquisition time.

**Figure 3 f3:**
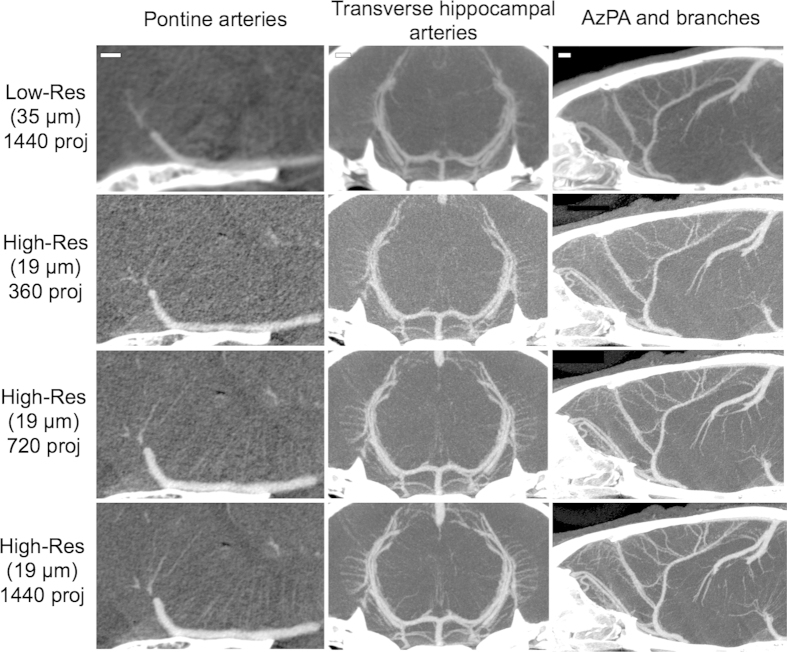
Effect of spatial resolution (voxel size) and projection number on vessel visibility. Representative thick slab maximum intensity projection images showing the effect of these parameters on pontine arteries, transverse hippocampal arteries and Azygos pericallosal artery (AzPA) and branches. Images are acquired at 50 kVp with a contrast agent dose of 2.2 gm I/kg. Scale bar represents 500 μm.

**Figure 4:**
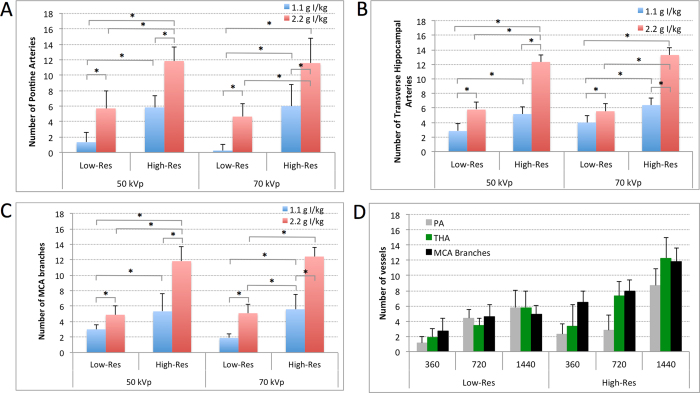
Quantitative analysis of the effect of scan parameters (peak voltage, spatial resolution and projection number) and contrast agent dose on vessel visibility . Analysis is performed on (**A**) pontine arteries (PA); (**B**) transverse hippocampal arteries (THA); and (**C**) middle cerebral artery branches (MCA branches). (**D**) Analysis of projection number (360, 720, 1440) is performed on scans acquired at 50 kVp and a contrast agent dose of 2.2 g I/kg. ^*^ *indicates statistically significant difference (p < 0.05)*.

Quantitative analysis of vessel number and vessel visibility sheds further insights into the effect of scan parameters. The visibility of sub-100 μm arteries is significantly impacted by image spatial resolution i.e., voxel size. Vessel visibility is quantified by counting the number of pontine arteries, transverse hippocampal arteries, and middle cerebral artery (MCA) branches visible in the images (Fig. 4). These vessels are assessed because (1) they cover a large portion of the brain vascular network (2) they have a range of vessel dimensions and (3) they are close to the skull which leads to interference of the images from the highly absorptive bone, therefore constituting a “worst case” scenario. In general, a larger number of vessels are seen in high-res images than in the low-res images. In the group receiving a low dose of contrast agent (1.1 g I/kg), a significantly higher number of pontine arteries are visible in high-res images compared to low-res images (Fig. 3,4A). The peak voltage (beam energy) does not have a significant impact on vessel visibility when comparing scans obtained with identical voxel size and contrast agent dose. Interestingly, in high-resolution scans at both peak voltage settings, the dose of the contrast agent does not significantly impact vessel visibility. Furthermore, a statistically significant dose-dependent increase in the number of visible vessels is demonstrated in high-res images for the transverse hippocampal arteries (Fig. 4B). These findings are particularly significant because concentric ring artifacts, which are common in micro-CT^40^,^41^, affect the visualization of the hippocampal arteries which have primarily a radial orientation. Vessel visibility analysis of the branches from the MCA also yields similar results as the transverse hippocampal arteries (Fig. 4C), with the largest number of MCA branches visible in high-res images acquired with a 2.2 g I/kg contrast agent dose.

In all cases, increased vessel visibility is observed in high-res images acquired with high contrast agent dose but the peak voltage did not alter vessel visibility (Supplementary Figure 5 and 6). Thus, a reduced peak voltage setting, and therefore a lower radiation dose, can generally be used without degrading image quality. The effect of the projection number on image quality was therefore studied in datasets acquired at 50 kVp and high contrast agent dose (2.2 g I/kg). The number of image projections significantly impacts visibility of blood vessels for all three vascular networks (Fig. 3,4). The number of vessels detectable increases almost two-fold in scans performed with 1440 projections compared to scans performed with 360 projections.

**Figure 5:**
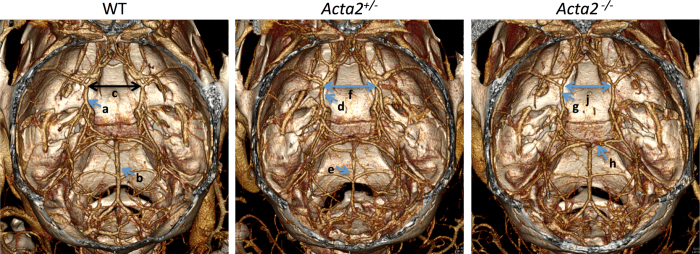
Coronal volume rendered images for 3D morphometric analysis of cerebral vasculature in mouse model of *Acta2* mutations. *Acta2*^*+/-*^ and *Acta2*^*-/-*^ mice manifests alterations in cerebral vasculature, demonstrated by narrowing of the Circle of Willis (**f,j**) and straightening of cerebral vessels (**d,g**) In addition, partial stenotic vessels are seen sporadically in *Acta2*^*+/-*^ and *Acta2*^*-/-*^ mice (**e,h**) None of the wild type (WT) mice had evidence of similar arterial narrowing (**c,a,b**).

High resolution imaging in conjunction with a high dose of contrast agent was used to study cerebral vascular structures in the wild type, *Acta2*^+/-^ and *Acta2*^-/-^ mice. Morphometric analysis was performed on the vessels within the Circle of Willis. Statistically significant differences (p < 0.05) are observed in the width of Circle of Willis between the wild type and knock-out mice (Fig. 5).The width of Circle of Willis is 2.82 ± 0.19 mm for the wild type mice, 2.74 ± 0.23 mm for the *Acta2*^*+/-*^ mice and 2.56 ± 0.08 mm for the *Acta2*^*-/-*^ group (wild type versus *Acta2*^*-/-*^ mice; p < 0.05). In addition, vessel straightening and vessel stenosis is observed in cerebral vascular network of *Acta2*^*+/-*^ and *Acta2*^*-/-*^ mutant mice, most significantly in the left internal carotid artery and right superior cerebellar artery, recapitulating findings in patients with *ACTA2* mutations (Supplementary Table 5 and 6; Supplementary Figure 8). The *Acta2*^+/-^ and *Acta2*^-/-^ mice had evidence of narrowing of arteries that was not present in the wild type mice (Fig. 5). These stenosis involved basilar artery or the arteries in the Circle of Willis. Thus, these results demonstrate the utility of the imaging technique for non-invasive, *in vivo* 3D vascular morphometric analysis of entire cerebral vascular networks. Specifically, vessel straightening and macroscopic changes in vascular network, such as width of the Circle of Willis, occur over large domains, are often deep inside tissue, and are easily distorted in ex vivo tissue processing, and therefore are almost impossible to reliably measure using current histological or optical imaging techniques.

## Discussion

The availability of rodent models of neurologic conditions resulting from cerebrovascular disease demands the development of novel imaging techniques that enable simple, reproducible and high-resolution *in vivo* imaging of the complete vasculature in small animals. Non-invasive, *in vivo* imaging of rodent cerebrovasculature presents unique challenges due to small size of the arteries and the presence of the skull. Optical techniques using fluorescent probes have been the most popular method, but usually require the installation of transparent windows in the skull and can only be used to visualize arteries near the surface due to absorption and scattering of light by tissue. Carefully selected near-infrared wavelengths and highly fluorescent probes permit some imaging through the skull without the need for windows, but still are limited to arteries near the surface of the brain (2-4mm)^12^. The methods described in this study provide a novel, reliable technique for high-resolution *in vivo* micro-CT imaging of the entire mouse cerebrovasculature.

Small vessel sizes (< 100 μm) necessitate the use of high spatial resolution (small voxel size) which in turn requires ways to reduce image noise while maintaining or increasing image contrast. Image noise increases exponentially with reduction in voxel size and vessel diameter^42^. In the current study, prolonged X-ray exposure time and a large number of image projections are used to improve SNR at high spatial resolution. The extended X-ray exposure time is also required due to the low X-ray tube current (0.5 mA maximum on a micro-CT vs. 500 mA on clinical CT scanner), a common limitation of commercially available small animal micro-CT scanners. Additionally, we found that a high dose of contrast agent is essential to achieve adequate SNR to visualize these small vessels. In order to maintain uniform and stable opacification of the vascular compartment for the duration of these long CT scans (~ 50 min), we used a long circulating, blood-pool liposomal-iodinated contrast agent that is cleared slowly from the circulation. *In vivo* studies using such long circulating blood pool contrast agents at similarly high dose levels have been previously demonstrated in a variety of rodent models but have not achieved similar spatial resolutions^24^,^35^,^43^,^44^,^45^. Other blood pool CT agents have also been developed for pre-clinical CT imaging but have generally not achieved sufficient opacification to capture the high resolution images demonstrated in this work^22^,^23^. Although not commonly accessible, customized angiography systems based on a synchrotron X-ray source have been utilized for vascular imaging at high spatial resolution^46^,^47^,^48^. These systems can generate rapid, high photon fluxes and therefore allow short scan times, also permitting acquisition of X-ray angiograms.

Even minimal animal movement, typically due to non-uniform anesthesia, can substantially impact image quality when imaging <100 μm vessel structures. Minor motion (~100 μm) would not be detected when images are acquired at relatively lower spatial resolution (>150 μm). To prevent such motion, a custom 3D mouse head holder was designed for this study. This holder insures uniform anesthetic flow into the lungs of the animal and practically eliminates unwanted motion (Supplementary Figures 1 and 2). This device enables reproducible visualization of complete mouse cerebral vasculature at high spatial resolution (19 μm). In fact, arteries as small as 40 μm in diameter are clearly visualized in the high-resolution CT angiograms.

X-ray imaging has been used to interrogate the rodent vasculature, primarily through *ex vivo* imaging. Dorr *et al.* used a combination of ex vivo high-resolution micro-CT and MRI to develop the CBA mouse vascular atlas^20^. Myojin *et al.*^49^ utilized synchrotron radiation microangiography for *ex vivo* imaging of mouse neurovasculature. Microspheres and barium sulfate microparticles were perfused to enhance the contrast in the blood vessels. Figueiredo *et al.*^50^ studied *in vivo* X-ray digital subtraction and CT angiography after administration of conventional contrast agent in an artery, simulating angiograms that are routinely performed in clinical practice. However, the latter approach is challenging to use for routine small animal imaging due to difficulty in reproducible arterial access in mouse models and a very narrow imaging window due to rapid clearance of conventional contrast agent. Furthermore, unlike small animal micro-CT, digital subtraction angiography systems are not readily available.

Since CT imaging involves the use of X-ray radiation, the radiation dose associated with micro-CT scans is an important factor in studies requiring longitudinal imaging. The effect of radiation dose on animal health has been studied using *in silico* models and *in vivo* in rodents^51^,^52^,^53^,^54^. The average LD_50/30_ radiation dose in mice from a single acute scan is reported as 7 Gy^54^. However, a high cumulative dose, given over a longer duration and in small fractions has relatively low impact on animal health^55^. Typical micro-CT examination, involving whole body imaging, results in a radiation dose of approximately 100 mGy. However, the majority of such micro-CT scan protocols utilize ≤ 400 X-ray projections and/or short durations of X-ray exposure. Micro-CT visualization of micron-sized vessel features, however, requires the use of high spatial resolution and therefore a correspondingly high radiation dose (higher X-ray exposure time and/or increased number of projections). Methods to reduce radiation dose (reduced kVp and image projections) were, therefore, implemented in our study. Studies involving assessment of pontine arteries or similar-sized structures (40 μm or smaller) would therefore require implementation of a 50 kVp or a 70 kVp scan protocol with small voxel size (19 μm) and 1440 image projections. Since the radiation dose associated with such a protocol is high (8-13 Gy), longitudinal imaging would be difficult. On the other hand, if the objective of the study is assessment of larger vessels, such as basilar arteries or major vessels within the Circle of Willis (i.e. 100 μm or larger), a 50 kVp scan protocol with large voxel size (35 μm) and 360 projections would be adequate, resulting in 14-24-fold reduction in radiation dose levels and therefore enabling longitudinal imaging studies. Although not investigated in this study, the use of advanced reconstruction algorithms, such as iterative and statistical reconstructions, is likely to further improve image quality^16^,^56^,^57^.

Implementing such methods enables the visualization of the complete cerebral vasculature in mice, with no spatial distortions such as those typically resulting from ex vivo studies. Furthermore, vessel diameters of major arteries within the Circle of Willis determined using imaging technique described in here are within the ranges reported previously in the literature^38^,^39^. It is therefore possible to evaluate morphological changes in the arteries over the entire organ. Therefore, we were able to demonstrate and quantify narrowing of the Circle of Willis and abnormal straightening of large vessels in *Acta2* mutant mice, along with stenosis of arteries. The straightening and stenosis in the *Acta2* mutant mice recapitulate findings in the humans with *ACTA2* mutations. Such distinct and significant changes would not be possible to assess without the imaging technique and quantification methods presented here. The narrowing of the Circle of Willis in cerebrovascular pathologies has not previously been reported. Our finding therefore signifies the importance of evaluating macro-morphometric changes in vascular network that goes beyond individual vessel assessment. These imaging protocols, therefore, allow for the non-invasive *in vivo* interrogation of rodent vasculature and open the door to studying the cerebrovascular disease initiation and progression in mouse models of human disease.

The techniques described here can be implemented for performing high-resolution imaging of extra-cranial vasculature, such as in the heart and abdomen. The long X-ray exposure period, used in our studies to achieve adequate SNR, may present challenges in gated-CT acquisitions, and therefore, result in longer acquisition times. The use of supplementary techniques, such as forced respiratory systems associated with locking triggered imaging acquisition, multiple-period averaging and compressed sensing methods may facilitate in overcoming these hurdles^16^,^57^,^58^.

## Conclusion

A method for high-resolution *in vivo* imaging of complete mouse cerebrovasculature using micro-CT is presented in this work. Blood vessels as small as 40 μm are clearly demonstrated in images acquired using the technique. The assessment of the *Acta2* mutant mice show that these techniques can successfully detect cerebrovascular disease in mouse models of human disease.

## Additional Information

**How to cite this article**: Starosolski, Z. *et al*. Ultra High-Resolution *In vivo* Computed Tomography Imaging of Mouse Cerebrovasculature Using a Long Circulating Blood Pool Contrast Agent.*Sci. Rep.* **5**, 10178; doi: 10.1038/srep10178 (2015).

## Supplementary Material

Supplementary Information

Supplementary Movie
