# Supplementary material for: Ultra High-Resolution In vivo Computed Tomography Imaging of Mouse Cerebrovasculature Using a Long Circulating Blood Pool Contrast Agent
Source: Sci Rep. 2015 May 18;5:10178. doi: 10.1038/srep10178 (PMC4650815; doi:10.1038/srep10178)
Supplement: Supplementary Information [file srep10178-s1.pdf]

Ultra High-Resolution *In vivo* Computed Tomography Imaging of Mouse Cerebrovasculature Using a Long Circulating Blood Pool Contrast Agent

Zbigniew Starosolski, Carlos A. Villamizar, David Rendon, Michael J. Paldino, Dianna M. Milewicz, Ketan B. Ghaghada, Ananth V. Annapragada

Supplementary Information

Table 1. Experimental matrix of parameters investigated in the study.

**Contrast agent dose (g Iodine/kg)**	1.1				2.2			
**Peak voltage (kVp)**	50		70		50		70	
**Voxel size (µm)**	19 (High-Res)	35 (Low-Res)	19 (High-Res)	35 (Low-Res)	19 (High-Res)	35 (Low-Res)	19 (High-Res)	35 (Low-Res)

Table 2. Radiation dose (Gy) as a function of peak voltage, number of projections and image resolution.

**No. of projections**	**50 kVp**		**70 kVp**	
	**Low-Res**	**High-Res**	**Low-Res**	**Low-Res**
**360**	0.54	1.93	0.93	3.27
**720**	1.09	3.85	1.86	6.54
**1440**	2.18	7.7	3.71	13.07

<u>Figure 1</u>: (A) 3D drawings of the bite bar and mouse head holder used for positioning of the animal in the micro-CT scanner. Both of the objects were 3D printed as per the details provided in the materials and method section. (B) Sagittal view demonstrating the supine positioning of the animal during CT image acquisition, insuring airway patency. The circular objects at the bottom are cylindrical iodine-containing phantoms placed in the field of view for image quality control.

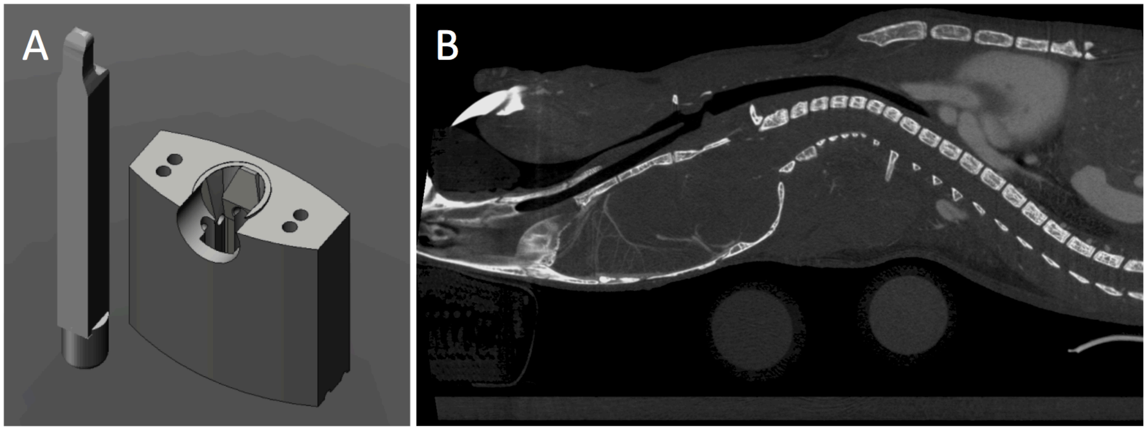

Figure 2. Effect of head holder on animal motion. The animal displays motion (yellow arrows) in the absence of head holder. Based on analysis of skull shift, the motion was determined to be 380 μm. The use of head holder helps minimize such motion. The airways are seen open when the animal is positioned in the head holder.

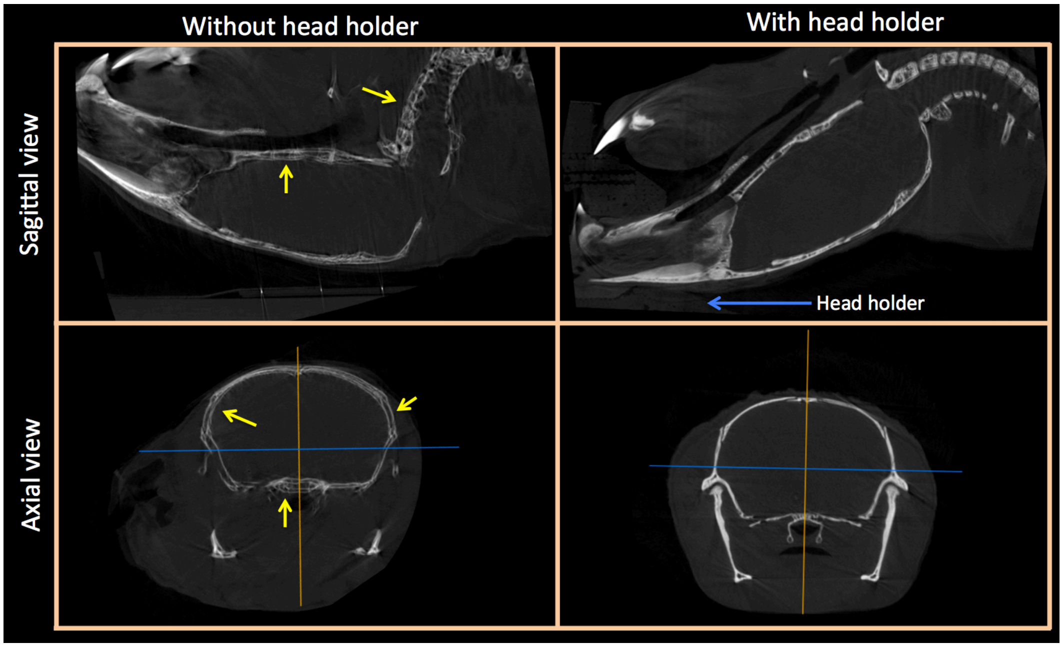

<u>Figure 3</u>: Placement of regions of interest (ROI) for determination of CT attenuation in the (A) internal carotid artery (ICA), and (B) great cerebral vein of Galen (GVG). Background ROI was drawn for determination of noise level, defined as standard deviation in the brain parenchyma, for use in the estimation of vessel SNR.

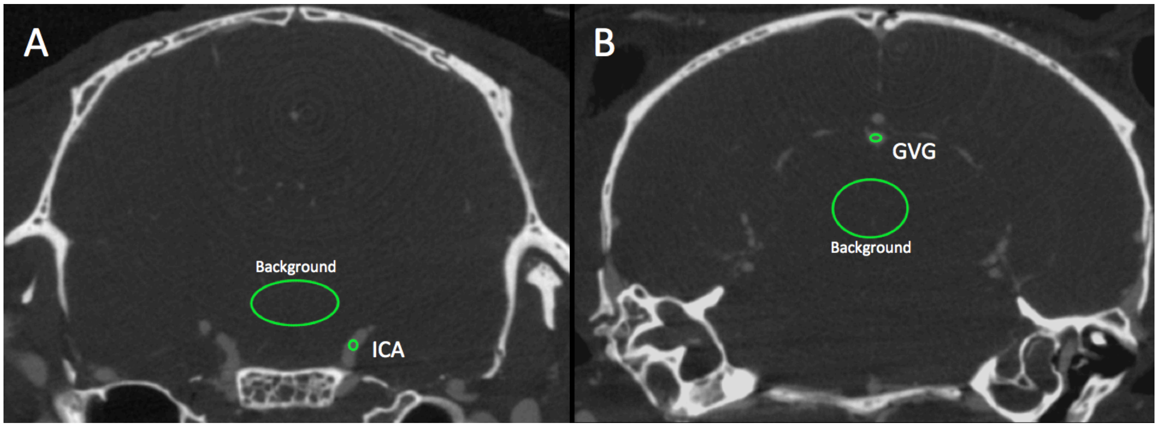

Figure 4. Quantitative analysis of vessel visibility. Coronal thick slab maximum intensity projection images demonstrating different grade levels for visibility of pontine arteries: Grade 0 – Figure A; Grade 1 – Figure B; Grade 2 – Figure C. 3D coronal volume rendered image of the mouse brain depicting middle cerebral artery (MCA) and its branches (Figure D). Branches marked with green dashed lines are sequentially numbered 1-11.

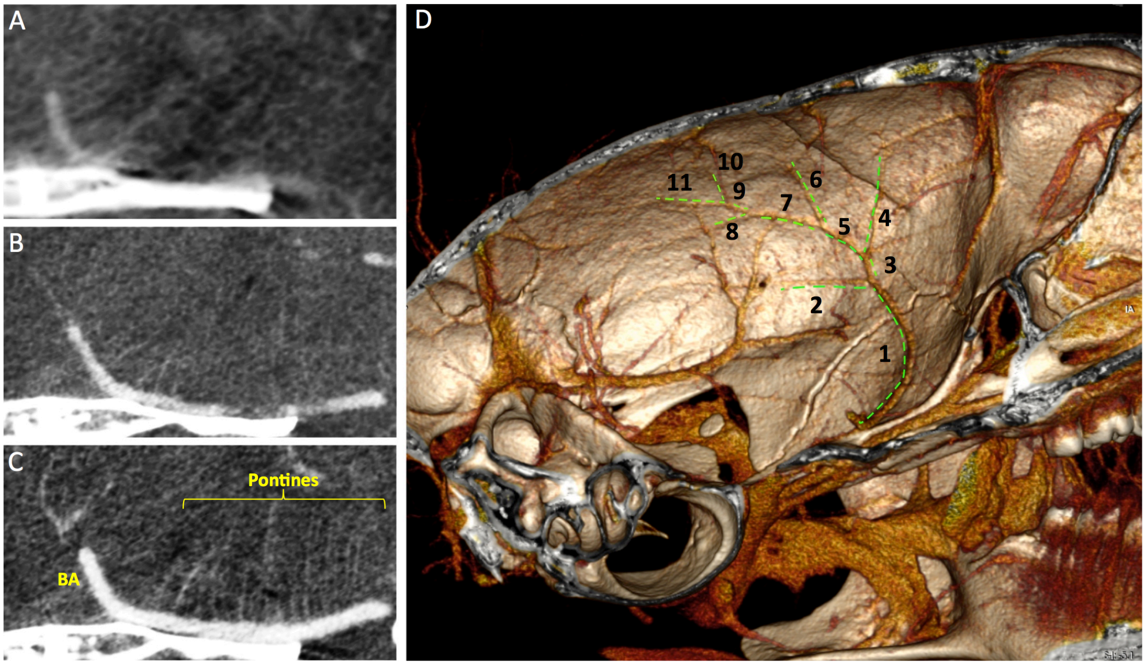

Statistical Analysis

Analysis of statistical significance was done by first testing for normal distribution of the data. The Kolmogorov–Smirnov non-parametric statistic was used to evaluate normality hypothesis of data distribution. P-values were calculated with a two-sided t-test if the data were normally distributed. Otherwise, statistical analysis was conducted using the two-sided Wilcoxon Rank-Sum test.

The effect of protocol parameters i.e., peak voltage (kVp), voxel size (spatial resolution) and contrast agent dose on calculated values of mean CT signal with region of interests, vessel diameters and visibility score were examined. The following hypotheses were tested:

a) Effect of spatial resolution (*Res*): high-resolution 19 µm (*HighRes*) and low-resolution 35 µm (*Low-Res*) on vessel diameter assessment (*d*).

$$\mathrm{H0}: d(LowRes) = d(HighRes),$$

b) Effect of contrast agent dose and peak voltage setting (kVp) on CT attenuation (Hounsfield Units, *HU*)

$$\mathrm{H0}:\ \mathrm{HU}x\ (kVp, dose) = \mathrm{HU}y(kVp, dose)$$

$$for \quad x, y\ \subset \{\mathrm{GVG}, \mathrm{ICA}\}, kVp \subset \{50{,}70\}, dose \subset \{1.1{,}2.2\}$$

c) Effect of contrast agent dose and peak voltage setting (*kVp)* setting on *SNR*.

$$\mathrm{H0}:\ \mathrm{SNRx}\ (kVp, dose) = \mathrm{SNRy}(kVp, dose)$$

$$for \quad x, y\ \subset \{\mathrm{GVG}, \mathrm{ICA}\}, kVp \subset \{50{,}70\}, dose \subset \{1.1{,}2.2\}$$

d) Effect of contrast agent dose and kVp setting on vessel visibility score (VesSCORE).

$$\mathrm{H0}:\ \mathrm{VesSCORE}x\ (kVp, dose, Res) = \mathrm{VesSCORE}y(kVp, dose, Res)$$

$$for \quad x, y\ \subset \{\mathrm{GVG}, \mathrm{ICA}\}, kVp \subset \{50{,}70\}, dose \subset \{1.1{,}2.2\}, Res \subset \{\mathrm{Low}, \mathrm{High}\}.$$

Figure 5: (A) Effect of peak voltage setting (kVp) and contrast agent dose on signal to noise ratio (SNR) for internal carotid artery (ICA) and great vein of Galen (GVG). (B) Two-sided Wilcoxon Rank-Sum test analysis of the comparative sub-groups. Entries in bold text represent statistical significance (p-value < 0.05).

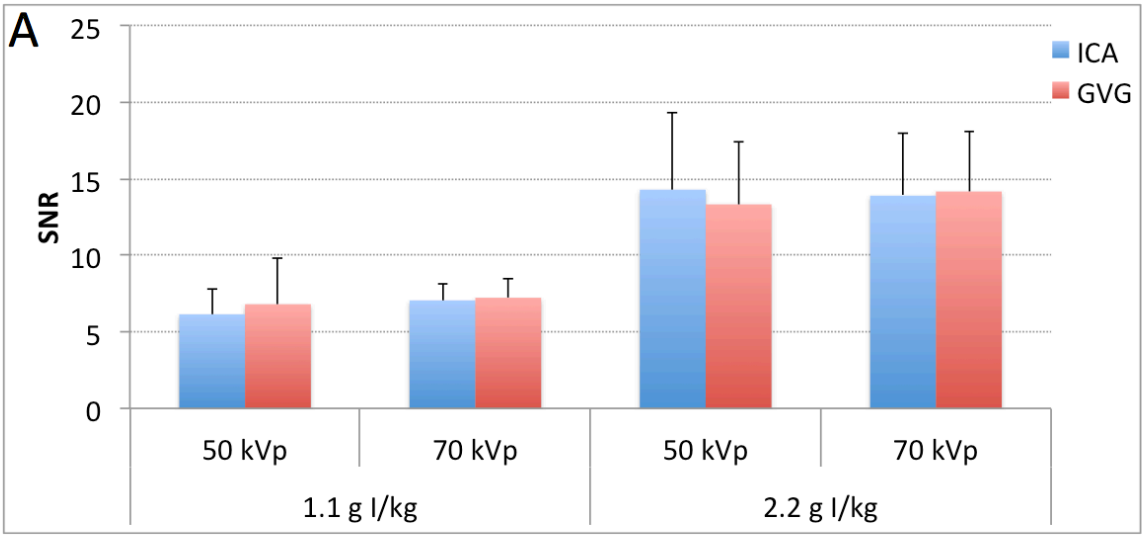

B

	(50 kVp, 1.1 g I/kg, ICA)	(70 kVp, 1.1 g I/kg, ICA)	(50 kVp, 1.1 g I/kg, GVG)	(70 kVp, 1.1 g I/kg, GVG)	(50 kVp, 2.2 g I/kg, ICA)	(50 kVp, 2.2 g I/kg, ICA)	(50 kVp, 2.2 g I/kg, GVG)	(70 kVp, 2.2 g I/kg, GVG)
(50 kVp, 1.1 g I/kg, ICA)	1.000							
(70 kVp, 1.1 g I/kg, ICA)	0.856	1.000						
(50 kVp, 1.1 g I/kg, GVG)	0.411	0.800	1.000					
(70 kVp, 1.1 g I/kg, GVG)	0.066	0.591	0.658	1.000				
(50 kVp, 2.2 g I/kg, ICA)	**<0.001**	**<0.001**	**<0.001**	**<0.001**	1.000			
(50 kVp, 2.2 g I/kg, ICA)	**<0.001**	**<0.001**	**<0.001**	**<0.001**	0.849	1.000		
(50 kVp, 2.2 g I/kg, GVG)	**<0.001**	**<0.001**	**<0.001**	**<0.001**	0.646	0.518	1.000	
(70 kVp, 2.2 g I/kg, GVG)	**<0.001**	**<0.001**	**<0.001**	**<0.001**	0.985	0.640	0.556	1.000

Figure 6: Effect of peak voltage (kVp), image voxel size and contrast agent dose on vessel conspicuity analysis for (A) pontine arteries and (B) transverse hippocampal arteries (B) Two-sided Wilcoxon Rank-Sum test analysis was performed on the sub-groups for the data presented in figure A and figure B, the results of which are presented in (C) and (D), respectively. Entries in bold text represent statistical significance (p-value < 0.05).

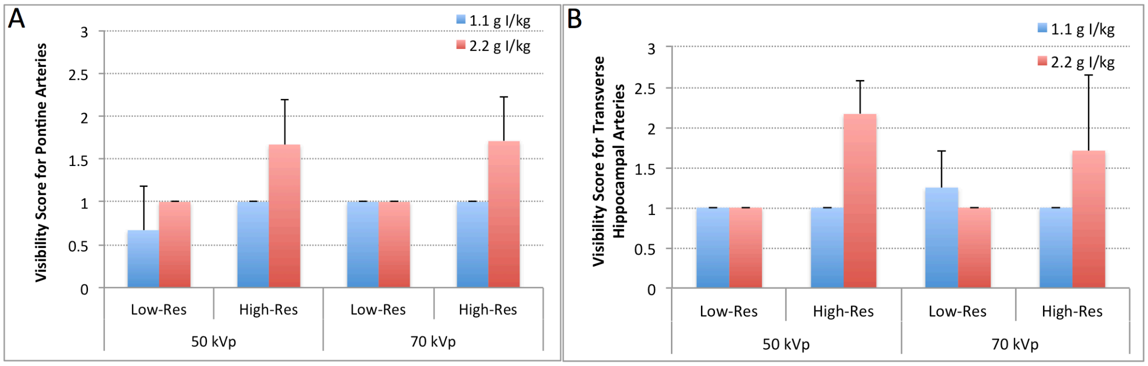

**C**	(50 kVp, 1.1 g I/kg, Low-Res)	(50 kVp, 2.2 g I/kg, Low-Res)	(50 kVp, 1.1 g I/kg, High-Res)	(50 kVp, 2.2 g I/kg, High-Res)	(70 kVp, 1.1 g I/kg, Low-Res)	(70 kVp, 2.2 g I/kg, Low-Res)	(70 kVp, 1.1 g I/kg, High-Res)	(70 kVp, 2.2 g I/kg, High-Res)
(50 kVp, 1.1 g I/kg, Low-Res)	1.000							
(50 kVp, 2.2 g I/kg, Low-Res)	0.059	1.000						
(50 kVp, 1.1 g I/kg, High-Res)	0.138	1.000	1.000					
(50 kVp, 2.2 g I/kg, High-Res)	**0.014**	**0.004**	**0.019**	1.000				
(70 kVp, 1.1 g I/kg, Low-Res)	**0.044**	**<0.001**	**0.002**	**0.001**	1.000			
(70 kVp, 2.2 g I/kg, Low-Res)	**0.048**	1.000	1.000	**0.003**	<0.001	1.000		
(70 kVp, 1.1 g I/kg, High-Res)	0.174	1.000	1.000	**0.029**	**0.003**	1.000	1.000	
(70 kVp, 2.2 g I/kg, High-Res)	**0.018**	0.188	**0.033**	0.735	**0.001**	0.170	**0.048**	1.000

**D**	(50 kVp, 1.1 g I/kg, Low-Res)	(50 kVp, 2.2 g I/kg, Low-Res)	(50 kVp, 1.1 g I/kg, High-Res)	(50 kVp, 2.2 g I/kg, High-Res)	(70 kVp, 1.1 g I/kg, Low-Res)	(70 kVp, 2.2 g I/kg, Low-Res)	(70 kVp, 1.1 g I/kg, High-Res)	(70 kVp, 2.2 g I/kg, High-Res)
(50 kVp, 1.1 g I/kg, Low-Res)	1.000							
(50 kVp, 2.2 g I/kg, Low-Res)	1.000	1.000						
(50 kVp, 1.1 g I/kg, High-Res)	1.000	1.000	1.000					
(50 kVp, 2.2 g I/kg, High-Res)	**0.001**	**<0.001**	**0.001**	1.000				
(70 kVp, 1.1 g I/kg, Low-Res)	0.202	0.103	0.202	**0.006**	1.000			
(70 kVp, 2.2 g I/kg, Low-Res)	1.000	1.000	1.000	**<0.001**	0.088	1.000		
(70 kVp, 1.1 g I/kg, High-Res)	1.000	1.000	1.000	**0.003**	0.243	1.000	1.000	
(70 kVp, 2.2 g I/kg, High-Res)	0.081	**0.028**	0.081	0.249	0.332	**0.021**	0.109	1.000

Figure 7: Effect of spatial resolution (voxel size) and projection number on image profiles of transverse hippocampal arteries. Axial images are presented as 1 mm MIP images acquired at 50 kVp with a contrast agent dose of 2.2 gm I/kg.

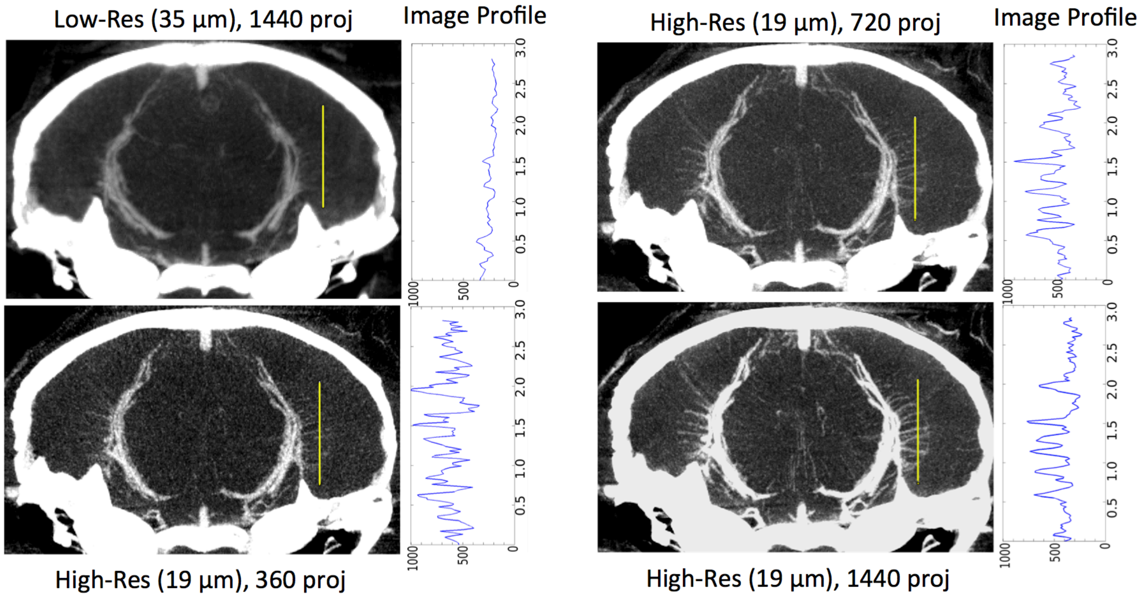

Additional results from statistical analysis of data presented in figures in the main article

Table 3. Statistical analysis of CT attenuation data presented in Figure 1A of main article. Entries in bold text represent statistical significance (p-value < 0.05).

	(50 kVp, 1.1 g I/kg, ICA)	(70 kVp, 1.1 g I/kg, ICA)	(50 kVp, 1.1 g I/kg, GVG)	(70 kVp, 1.1 g I/kg, GVG)	(50 kVp, 2.2 g I/kg, ICA)	(50 kVp, 2.2 g I/kg, ICA)	(50 kVp, 2.2 g I/kg, GVG)	(70 kVp, 2.2 g I/kg, GVG)
(50 kVp, 1.1 g I/kg, ICA)	1.000							
(70 kVp, 1.1 g I/kg, ICA)	**0.036**	1.000						
(50 kVp, 1.1 g I/kg, GVG)	0.248	0.497	1.000					
(70 kVp, 1.1 g I/kg, GVG)	0.384	0.412	0.109	1.000				
(50 kVp, 2.2 g I/kg, ICA)	**<0.001**	**<0.001**	**<0.001**	**<0.001**	1.000			
(50 kVp, 2.2 g I/kg, ICA)	**0.002**	**<0.001**	**0.011**	**0.001**	**0.040**	1.000		
(50 kVp, 2.2 g I/kg, GVG)	**<0.001**	**<0.001**	**<0.001**	**<0.001**	0.885	**0.036**	1.000	
(70 kVp, 2.2 g I/kg, GVG)	**<0.001**	**<0.001**	**0.001**	**<0.001**	0.093	0.438	0.050	1.000

Table 4. Statistical analysis of data on effect of projection number on vessel visibility presented in Figure 4D of main article. (A) Pontine arteries, (B) Transverse hippocampal arteries, (C) MCA branches. Entries in bold text represent statistical significance (p-value < 0.05).

**A**	(High-Res, 1440 projs)	(High-Res, 720 projs)	(High-Res, 360 projs)	(Low-Res, 1440 projs)	(Low-Res, 720 projs)	(Low-Res, 360 projs)
(High-Res, 1440 projs)	1.000					
(High-Res, 720 projs)	**0.043**	1.000				
(High-Res, 360 projs)	**0.004**	**0.010**	1.000			
(Low-Res, 1440 projs)	0.064	0.857	**0.014**	1.000		
(Low-Res, 720 projs)	**0.003**	0.149	**0.010**	0.128	1.000	
(Low-Res, 360 projs)	**0.001**	**0.001**	0.056	**0.001**	**<0.001**	1.000

**B**	(High-Res, 1440 projs)	(High-Res, 720 projs)	(High-Res, 360 projs)	(Low-Res, 1440 projs)	(Low-Res, 720 projs)	(Low-Res, 360 projs)
(High-Res, 1440 projs)	1.000					
(High-Res, 720 projs)	**0.015**	1.000				
(High-Res, 360 projs)	**0.004**	**0.030**	1.000			
(Low-Res, 1440 projs)	**0.001**	0.257	0.106	1.000		
(Low-Res, 720 projs)	**0.001**	**0.002**	0.674	**0.023**	1.000	
(Low-Res, 360 projs)	**0.001**	**0.001**	0.304	**0.002**	**0.010**	1.000

**C**	(High-Res, 1440 projs)	(High-Res, 720 projs)	(High-Res, 360 projs)	(Low-Res, 1440 projs)	(Low-Res, 720 projs)	(Low-Res, 360 projs)
(High-Res, 1440 projs)	1.000					
(High-Res, 720 projs)	**0.008**	1.000				
(High-Res, 360 projs)	**0.004**	0.101	1.000			
(Low-Res, 1440 projs)	**0.001**	**0.003**	**0.034**	1.000		
(Low-Res, 720 projs)	**0.001**	**0.002**	**0.034**	0.927	1.000	
(Low-Res, 360 projs)	**0.001**	**0.001**	**0.003**	**0.014**	**0.024**	1.000

Vascular morphometric analysis of ACTA2 knock-out mouse models

The following vascular morphometric parameters were calculated from the cerebrovascular CT images acquired in mouse models of moyamoya disease:

- Radius (mm): Median value calculated from a set of minimal radius of segments, (all segments combined together represent the vessel under examination).
- Length (mm): Length of centerline of a blood vessel.
- Middle curvature (mm^{-1}): For a centerline C and a middle point P on it, there is a unique circle or line that most closely approximates the curve near P. Curvature is expressed as the inverse of the circle radius.
- Arc:length Ratio: Also referred to as tortuosity, is calculated as the length along centerline divided by Euclidean distance between beginning and end of centerline.
- Narrowing (%): Number of segments that are smaller than 85% of the median radius value.
- Mean: Mean aspect ratio is defined as a ratio between first and second eigenvalues calculated for contour for one point along centerline. Mean is calculated for a set of separated aspect ratios.

Table 5. Statistical analysis on vascular morphometric parameters of cerebrovascular arteries determined by CT imaging. In all cases, null hypotheses H_0: WT = *Acta2*$^{+/-}$, H_0: WT = *Acta2*$^{-/-}$, H_0: *Acta2*$^{+/-}$ = *Acta2*$^{-/-}$ were tested. The two-sided Wilcoxon Rank-Sum test was used to assess statistical significance. Bolded entries indicate measures where the null hypothesis was disproven at the 95% confidence level.

Vessel	Radius	Length	Middle Curvature	Arc:Length	Narrowing	Mean
**Right Internal Carotid**						
WT vs. *Acta2*$^{+/-}$	0.536	0.607	0.837	0.470	0.719	0.174
Acta2$^{+/-}$ vs. *Acta2*$^{-/-}$	**0.005**	0.689	0.689	0.328	0.709	0.114
WT vs. *Acta2*$^{-/-}$	**0.014**	0.945	0.445	0.101	0.333	0.945
**Right Posterior Cerebral**						
WT vs. *Acta2*$^{+/-}$	0.470	1.000	0.211	0.536	0.381	0.837
Acta2$^{+/-}$ vs. *Acta2*$^{-/-}$	0.272	0.689	0.864	0.864	**0.038**	0.328
WT vs. *Acta2*$^{-/-}$	0.366	0.731	0.295	0.366	**0.005**	0.534
**Right Superior Cerebellar**						
WT vs. *Acta2*$^{+/-}$	0.681	0.211	0.681	0.114	0.527	1.000
Acta2$^{+/-}$ vs. *Acta2*$^{-/-}$	0.088	0.145	**0.018**	**0.026**	0.896	0.955
WT vs. *Acta2*$^{-/-}$	0.138	0.073	**0.023**	**0.008**	0.457	0.731
**Right Anterior Cerebral**						
WT vs. *Acta2*$^{+/-}$	0.299	0.252	0.837	0.837	0.448	0.837
Acta2$^{+/-}$ vs. *Acta2*$^{-/-}$	0.066	0.272	0.607	0.388	0.208	0.388
WT vs. *Acta2*$^{-/-}$	0.445	0.534	0.295	0.366	0.469	**0.001**
**Left Internal Carotid**						
WT vs. *Acta2*$^{+/-}$	0.758	0.071	**0.003**	**0.012**	0.988	**0.023**
Acta2$^{+/-}$ vs. *Acta2*$^{-/-}$	**0.018**	0.456	**0.050**	0.272	0.269	0.864
WT vs. *Acta2*$^{-/-}$	**0.022**	**0.022**	**0.001**	**0.014**	0.347	0.138
**Left Posterior Cerebral**						
WT vs. *Acta2*$^{+/-}$	0.071	**0.031**	**0.002**	**0.005**	0.197	0.252
Acta2$^{+/-}$ vs. *Acta2*$^{-/-}$	0.145	0.181	0.145	0.066	0.168	0.776
WT vs. *Acta2*$^{-/-}$	0.534	0.295	0.101	0.836	0.977	0.445
**Left Superior Cerebellar**						
WT vs. *Acta2*$^{+/-}$	0.408	0.758	0.681	0.536	0.908	0.681
Acta2$^{+/-}$ vs. *Acta2*$^{-/-}$	0.145	0.607	0.955	0.388	0.544	0.776
WT vs. *Acta2*$^{-/-}$	0.628	0.945	0.945	0.101	0.977	0.445
**Left Anterior Cerebral**						
WT vs. *Acta2*$^{+/-}$	1.000	0.299	0.408	0.536	0.416	1.000
Acta2$^{+/-}$ vs. *Acta2*$^{-/-}$	0.328	0.456	0.955	0.864	0.641	0.224
WT vs. *Acta2*$^{-/-}$	0.234	1.000	0.295	0.731	0.918	0.445

Figure 8: A schematic of the Circle of Willis describing the vascular morphometric parameters.

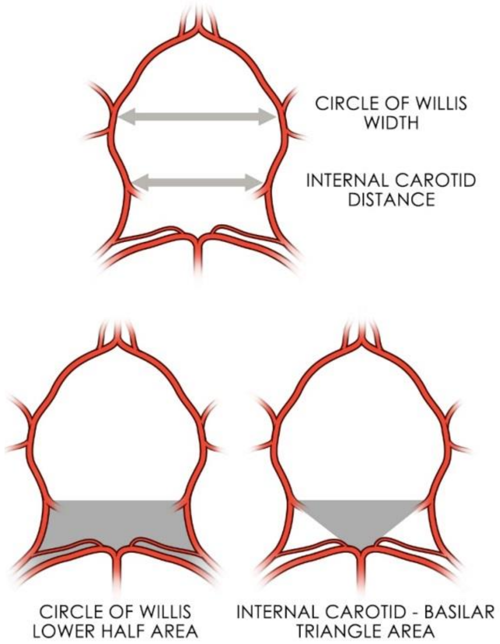

Table 6. Statistical analysis of the dimensions in the Circle of Willis determined by CT imaging. For the total area, upper half area and width, the null hypothesis H_0: WT = *Acta2*$^{+/-}$, H_0: WT = *Acta2*$^{-/-}$, H_0: *Acta2*$^{+/-}$ = *Acta2*$^{-/-}$ were tested. In all cases, the two-sided Wilcoxon Rank-Sum test was used to assess statistical significance. Bolded entries indicate measures where the null hypothesis was disproven at the 95% confidence level.

Circle of Willis	Total Area	Upper Half Area	Lower Half Area	Basal Triangle Area	Width	Narrowing % of all COW vessels
WT vs. *Acta2*$^{+/-}$	**0.023**	0.918	**0.008**	**0.012**	0.351	0.270
Acta2$^{+/-}$ vs. *Acta2*$^{-/-}$	0.456	0.088	0.529	0.607	0.388	0.473
WT vs. *Acta2*$^{-/-}$	0.445	0.101	**0.005**	**0.005**	**0.002**	0.043
